# The Swiss cheese model of safety incidents: are there holes in the metaphor?

**DOI:** 10.1186/1472-6963-5-71

**Published:** 2005-11-09

**Authors:** Thomas V Perneger

**Affiliations:** 1 Institute of Social and Preventive Medicine, University of Geneva, Geneva, Switzerland; 2 Quality of Care Service, University Hospitals of Geneva, CH-1211 Geneva 14, Switzerland

## Abstract

**Background:**

Reason's Swiss cheese model has become the dominant paradigm for analysing medical errors and patient safety incidents. The aim of this study was to determine if the components of the model are understood in the same way by quality and safety professionals.

**Methods:**

Survey of a volunteer sample of persons who claimed familiarity with the model, recruited at a conference on quality in health care, and on the internet through quality-related websites. The questionnaire proposed several interpretations of components of the Swiss cheese model: a) slice of cheese, b) hole, c) arrow, d) active error, e) how to make the system safer. Eleven interpretations were compatible with this author's interpretation of the model, 12 were not.

**Results:**

Eighty five respondents stated that they were very or quite familiar with the model. They gave on average 15.3 (SD 2.3, range 10 to 21) "correct" answers out of 23 (66.5%) – significantly more than 11.5 "correct" answers that would expected by chance (p < 0.001). Respondents gave on average 2.4 "correct" answers regarding the slice of cheese (out of 4), 2.7 "correct" answers about holes (out of 5), 2.8 "correct" answers about the arrow (out of 4), 3.3 "correct" answers about the active error (out of 5), and 4.1 "correct" answers about improving safety (out of 5).

**Conclusion:**

The interpretations of specific features of the Swiss cheese model varied considerably among quality and safety professionals. Reaching consensus about concepts of patient safety requires further work.

## Background

**Figure 1:**
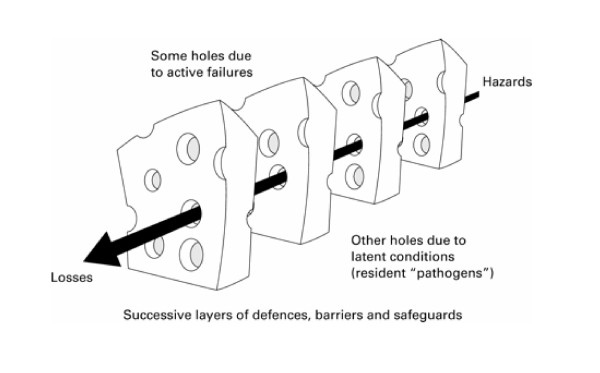
Swiss cheese model by James Reason published in 2000 (1). Depicted here is a more fully labelled black and white version published in 2001 (5). On the survey questionnaire, all labels and comments were hidden.

James Reason proposed the image of "Swiss cheese" to explain the occurrence of system failures, such as medical mishaps [1-5]. According to this metaphor, in a complex system, hazards are prevented from causing human losses by a series of barriers. Each barrier has unintended weaknesses, or holes – hence the similarity with Swiss cheese. These weaknesses are inconstant – i.e., the holes open and close at random. When by chance all holes are aligned, the hazard reaches the patient and causes harm (Figure 1). This model draws attention to the health care system, as opposed to the individual, and to randomness, as opposed to deliberate action, in the occurrence of medical errors.

The Swiss cheese model is frequently referred to and widely accepted by patient safety professionals. This was summarised by safety expert Ronald Westrum in a testimony before a United States Advisory Committee on Blood Safety and Availability on April 25, 2000 [6]:

"Reason's model has become the common language through which complex accidents can be understood. I remember being at one conference where six speakers in a row got up and showed Swiss cheese diagrams as a kind of academic overkill. The popularity of this model obviously comes from its wide application. It's generally felt, as I said, this provides a common ground for discussing system safety."

There is no clear evidence, however, that the Swiss cheese metaphor is understood in the same way by all concerned. In this study, I explored the understanding of the Swiss cheese model by professionals who work in healthcare quality improvement.

## Methods

### Samples and data collection

The data for this cross-sectional survey came from two sources: paper questionnaires filled by conference delegates, and online questionnaires. A self-completed questionnaire ("the Swiss cheese quiz") was handed out to attendees of the 20^th ^conference of the International Society for Quality in Health Care (Amsterdam, October 19–22, 2004), at the booth of the International Journal for Quality in Health Care. Completed questionnaires were collected in a ballot box. The same questionnaire was also posted on the internet [7]. Links to this site were placed on the access page of the International Journal for Quality in Health Care and the home page of the International Society for Quality in Health Care between November 2004 and January 2005.

### Questionnaire

The questionnaire displayed the picture of the Swiss cheese model, as published in the BMJ [1], but with the words "hazards" and "losses" hidden. The figure was followed by this statement: "As with many metaphors, there are several ways of interpreting this model. We would like to know your own interpretation. There are no right or wrong answers." The first questions probed the familiarity of the respondent with the model and its perceived usefulness. Further questions addressed the interpretation of various aspects of the Swiss cheese model: what is represented by a slice of cheese, by a hole, and by the arrow, how is an active error represented, and how would one make the system safer (Table 1). Each question was followed by 5 statements, and respondents were asked to check all that applied. Answer statements were classified a priori by the author as being compatible or incompatible with the model (Table 1). There were 11 compatible statements and 12 incompatible statements. Two additional statements were initially rated as incompatible with the model, but this was revised to "ambiguous" following advice from other experts: a) a health care professional could indeed be considered as a barrier if her or his role was primarily to prevent the occurrence of errors or of patient harm, and b) the arrow would represent the series of events leading to a medical error if the error was concomitant with patient harm. The questionnaire ended with questions about descriptive characteristics of the respondents.

**Table 1:** Interpretation of the Swiss cheese model of medical error by 85 professionals who claimed to be fairly or very familiar with the model.

	Compatibility with Swiss cheese model	N (%) endorsing statement	Percent "correct" answers
In your opinion, what does a slice of cheese represent?			
A health care professional	Sometimes^3^	14 (16.5)	-
A barrier that protects patients from harm	yes	61 (71.8)	71.8
A root cause of an error	no	9 (10.6)	89.4
A procedure that alleviates the consequences of an error	yes	14 (16.5)	16.5
A defence that prevents the occurrence of an error	yes	52 (61.2)	61.2

In your opinion, what does a hole represent?			
A latent error^1^	yes	28 (32.9)	32.9
A loss (in terms of health or money) due to an error	no	5 (5.9)	94.1
An opportunity for error	yes	53 (62.4)	62.4
A weakness in defences against error	yes	54 (63.5)	63.5
An unsafe act	yes	17 (20.0)	20.0

What does the arrow represent?			
The patient's trajectory through the health care system	no	29 (34.1)	65.9
A transfer of energy that injures a patient	no	2 (2.4)	97.6
The transformation of a latent error^1 ^into an active error^2^	no	24 (28.2)	71.8
The series of events leading to a medical error	Sometimes^4^	51 (60.0)	-
The path from hazard to patient harm	yes	41 (48.2)	48.2

How or where is an active error represented on this figure?			
At the base (origin) of the arrow	no	10 (11.8)	88.2
At the tip of the arrow	no	24 (28.2)	71.8
As one of the holes	yes	26 (30.6)	30.6
As the arrow itself	no	24 (28.2)	71.8
As the alignment of holes	no	28 (32.9)	67.1

How can we make the health care system safer, using the "Swiss cheese" metaphor?			
By adding a slice of cheese	yes	27 (31.8)	31.8
By removing a slice of cheese	no	6 (7.1)	92.9
By plugging a hole	yes	76 (89.4)	89.4
By adding a hole	no	1 (1.2)	98.8
By making all slices thinner	no	6 (7.1)	92.9

^1 ^Latent error: Failure of system design that increases the probability of harmful events. Loosely equivalent to causal factor or contributing factor.

^2 ^Active error: Error (of commission or omission) committed at the interface between a human and a complex system.

^3 ^A professional whose role is to make the process of care safer may be thought of as a protective barrier

^4 ^This would be true if the error equates with patient harm, as in the case of wrong site surgery

### Analysis

Because the goal was to explore the understanding of respondents who believed that they knew the model, only respondents who said they were "very much" or "quite a bit" familiar with the model were analysed. There was no statistically significant difference between those who filled the questionnaire at the conference and those who filled it online, thus the samples were pooled for the analysis.

The analysis consisted of simple frequencies of endorsement for each proposed answer. Endorsement of an item that was compatible with Reason's model according to the author, and non-endorsement of an incompatible item, were treated as "correct" answers. Sums of endorsed compatible and incompatible items were computed, as well as the number of "correct" answers out of 23. The two ambiguous items were left out of this analysis.

## Results

### Sample characteristics

Forty-eight usable questionnaires were collected at the conference (4 others were incomplete), and 111 on the internet (11 others were empty, duplicated, only partially filled, included only "I have no idea" answers, or had all answer options checked). Eighty-five respondents (53.5%) stated that they were "very" (N = 45) or "quite" (N = 40) familiar with the Swiss cheese model. Only these respondents are reported on in the analyses.

Participants were 44 years old on average (SD 9, range 25 to 70, 5 missing), and comprised 42 women and 42 men (1 missing), from 31 countries representing all continents. Most job titles were in health care policy and quality management, but respondents included also health care professionals (doctors, nurses and pharmacists) and academic researchers. Sixty-three (74.1%) respondents had worked in quality management in the past 5 years, 31 (36.9%) in risk management, and 19 (22.6%) in safety science (several answers were allowed; one person did not answer).

Most respondents thought that the Swiss cheese model was "very useful" (44, 51.8%) or "quite useful" (32, 37.6%). At the end of the questionnaire, 9 (10.7%) rated themselves as very knowledgeable about patient safety, 63 (75.0%) as quite knowledgeable, and 12 (14.3) as only a little knowledgeable.

### Interpretation of the model

Respondents endorsed on average 5.3 of 11 statements that were compatible with the Swiss cheese model (SD 2.2, range 1 to 10), and 2.0 of the 12 statements that were incompatible (SD 1.7, range 0 to 9). The mean number of "correct" answers was 15.3 (SD 2.3, range 10 to 21) out of 23 (66.5%). This was significantly more than 11.5 "correct" answers that would expected if answers were given at random (p < 0.001). Respondents gave on average 2.4 "correct" answers regarding the slice of cheese (out of 4), 2.7 "correct" answers about the holes (out of 5), 2.8 "correct" answers about the arrow (out of 4), 3.3 "correct" answers about the active error (out of 5), and 4.1 "correct" answers about improving safety (out of 5). None of the following variables were associated with the mean number of "correct" answers: sex, age, previous experience in quality management, risk management or safety science, familiarity with the model (very familiar versus quite familiar), perceived usefulness of the model, and knowledge of patient safety.

### Specific items

Most respondents interpreted the slice of cheese as intended by J. Reason (barrier that protects patients from harm), and inferred correctly that this would include a defence that prevents the occurrence of an error (Table 1). However, only few recognised that procedures that alleviate consequences of an error may also appear as barriers. Majorities interpreted a hole as suggested by Reason – a weakness in defences, but only few respondents understood that a hole is either a latent error or an unsafe act. The most obvious interpretation of the arrow (path from hazard to harm) was chosen by only half of the respondents. The majority choice (series of events leading to an error) is not entirely correct, as it is patient harm, not an error, that is represented by Reason at the tip of the arrow (however, the error may be equivalent to patient harm, as in wrong site surgery). Only three out of ten respondents identified correctly an active error as one of the holes. Making the system safer by plugging a hole was correctly selected by most respondents, but the solution of adding a barrier (a slice of cheese) was not.

## Discussion

This survey shows that among quality improvement professionals, the meaning of the Swiss cheese model of medical error is far from univocal. On average, respondents gave answers that were compatible with the model to about two thirds of the proposed statements. This is better than half – the proportion that would be expected by chance – but far from a general consensus. This suggests that invoking the Swiss cheese model will not necessarily lead to effective communication, even among quality and safety professionals.

There was substantial variability among respondents as to what the various features of the model represent. The murkiest notion appeared to be the representation of the medical error itself. Few of the respondents recognised that an active error is a type of weakness in defences against patient harm within the health care system, represented by a hole in the Swiss cheese model (a "hole" is either an active or a latent error). The model is almost too successful in placing emphasis on systemic causes of patient harm, as opposed to an individual's failure.

**Figure 2:**
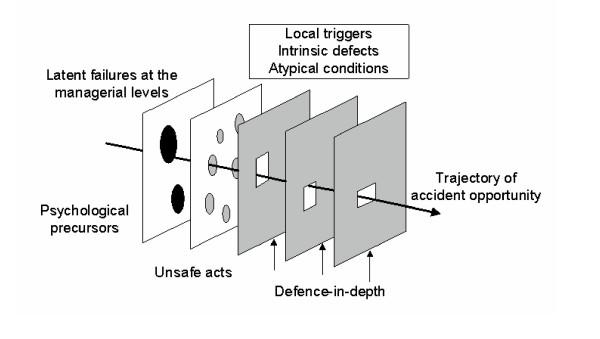
Reason's model published in 1990 (2).

**Figure 3:**
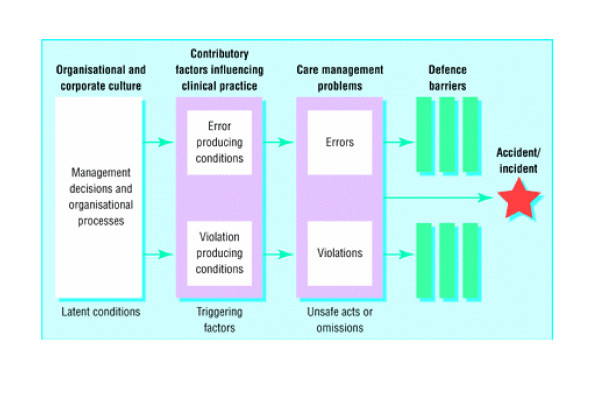
Reason's model published in 1995 (3), as adapted by Vincent et al (10).

**Figure 4:**
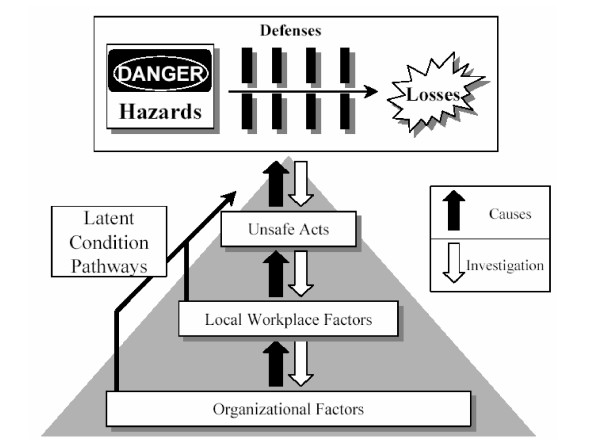
Reason's model published in 1997 (4).

The variability in interpretations revealed in this survey is more understandable if one considers the evolution of Reason's model between 1990 and 2000. In the first rendition of the model (Figure 2), what was predicted was an accident, latent errors were placed as antecedents of the accident trajectory at the far left, and unsafe acts (i.e., active errors) were represented by a separate "slice" [2]. Two subsequent models place the set of barriers between harm and the patient (the "slices of Swiss cheese") in a more global context (Figure 3 and 4). In particular, these models attempt to show causal chains that lead up to patient harm. For the sake of clarity, it should be noted that these more complete models have not been dubbed "Swiss cheese models." The model of 1995 (Figure 3) shows a sequence of conditions and events leading to an accident, and defences and barriers are represented as intervening only after the occurrence of an error or a violation [3]. The model published in 1997 (Figure 4) depicts the Swiss cheese model as leading to human losses, not accidents [4]. This supports the view that patient safety interventions should focus on patient harm, rather than errors [8,9]. Importantly, the 1997 model also displays unsafe acts and workplace factors as orthogonal to the arrow leading from hazards to losses – presumably, each weakness in the system has its own set of causal or contributing factors. The current version of the Swiss cheese model (Figure 1), published in 2000, appears to be a simplification of the previous model, from which the causal pathways have been removed.

While no model ever claims to represent fairly a complex reality, it is possible that the latest rendition of the Swiss cheese model has become too simplified to remain effective in promoting patient safety. More realistic alternatives include the model of 1995 (Figure 3), which clearly separates the event, or accident, from patient harm, and has remained in use [10], and that of 1997 (Figure 4), which suggests that the occurrence of a system failure cannot be easily represented by a simple linear sequence. In this Reason's model rejoins Haddon's matrix, a successful epidemiologic model for investigating injuries [11]. The relevance of Haddon's matrix for investigating medical mishaps has been recognised by others [8]. The integration of Reason's and Haddon's models may be a worthwhile next step toward a comprehensive model of patient safety.

More generally, the diversity of views documented in this study raises questions about the current status of a "culture of safety" among quality and safety professionals. A culture is a set of values, concepts and beliefs that are shared by a social group [12,13]. The Swiss cheese model is the leading candidate for a common understanding of how harmful events occur and how they can be prevented. Until most or all actors agree on what the model means, the emergence and dissemination of a shared culture of safety may prove difficult. The danger is that people today use the label "Swiss cheese model" without realising that its meaning varies from one person to the next.

This study has several limitations. The main concern is that the sample of respondents was self-selected, and may not represent fairly the broader community of patient safety and healthcare quality professionals. It is likely that those who were most interested and most knowledgeable about patient safety are over-represented among participants. Secondly, it is possible that respondents misrepresented their level of familiarity with the model, and that results would have been better among true experts. Nevertheless, the discrepancy between self-perceived familiarity with the model and variable interpretations of the model features is striking. Finally, the questionnaire was developed *ad hoc*, and its reliability and validity are untested.

In summary, this study has shown that quality and safety professionals vary considerably in their interpretation of various components of the Swiss cheese model applied to medical mishaps. This finding echoes the variability in interpretation that exists even for basic terms of patient safety, such as "incident," "error," "mishap," etc. [14]. Recent proposals of a comprehensive taxonomy of patient safety illustrate the necessity of a global conceptual model [15]. Good models and clear concepts are required for a common terminology, and a common terminology is a pre-requisite for effective communication and progress in the field of patient safety.

## Competing interests

The author(s) declare that they have no competing interests.

## Authors' contributions

The author conceived the study, pre-tested the questionnaire, collected and analysed the data, and wrote the paper.

## Pre-publication history

The pre-publication history for this paper can be accessed here:


